# A Rare Case of Epstein-Barr Virus: Infectious Mononucleosis Complicated by Guillain-Barré Syndrome

**DOI:** 10.7759/cureus.21085

**Published:** 2022-01-10

**Authors:** Oluseyi Abidoye, Erine Raybon-Rojas, Henry Ogbuagu

**Affiliations:** 1 Internal Medicine, Northeast Georgia Medical Center Gainesville, Gainesville, USA; 2 Critical Care Medicine, Northeast Georgia Medical Center Gainesville, Gainesville, USA

**Keywords:** epstein-barr virus, plasmapheresis, neuromuscular diseases, intravenous immunoglobulin (ivig), guillain-barré syndrome, infectious mononucleosis

## Abstract

Infectious mononucleosis (IM) is an acute disease caused by Epstein-Barr virus (EBV) infection affecting adolescents and young adults. Clinically, IM presents with fever, lymphadenopathy, and tonsillar pharyngitis. Guillain-Barré syndrome (GBS) has been reported as a possible rare complication of IM. IM-induced GBS is known but rarely reported in the literature. Here, we describe the case of a 19-year-old male with no significant medical history who was diagnosed with GBS following EBV-associated IM.

A 19-year-old Caucasian male presented from a referring facility after complaining of generalized weakness involving the upper and lower extremity for about five days. Symptoms began with a sensation of tingling and numbness in the fingertips and toes that progressed over five days to where he was no longer able to ambulate. Physical examination was significant for oropharyngeal exudates, posterior oropharyngeal erythema, tonsillar hypertrophy, cervical lymphadenopathy, flaccid paralysis with areflexia, and paresthesia. Diagnostic workup was consistent with IM and GBS based on cerebrospinal findings. He was subsequently admitted to the intensive care unit, where he received plasmapheresis and intravenous immunoglobulin with significant improvement. This is a rare case of EBV-associated IM GBS. IM is a self-limiting disease but can lead to GBS as one of the known but rare complications. Neurological events have been reported in approximately 2% of patients. Only a few cases of IM leading to GBS have been reported in the literature. Detailed history and physical examination can help identify patients with IM-induced GBS. Moreover, increased awareness can help physicians easily identify and manage GBS, enabling timely recognition and initiation of prompt supportive care to improve recovery time.

## Introduction

Infectious mononucleosis (IM) is an acute disease caused by Epstein-Barr virus (EBV) infection, which affects adolescents and young adults. The clinical presentation of IM includes fever, lymphadenopathy, and tonsillar pharyngitis [1]. Neurologic complications have been reported with IM, including cranial nerve palsies [2], encephalitis [3], aseptic meningitis, transverse myelitis, peripheral neuritis, optic neuritis, encephalomyelitis, and, rarely, Guillain-Barré syndrome (GBS) [4]. IM-induced GBS is rarely reported in the literature. Here, we describe the case of a 19-year-old male with no significant medical history who was diagnosed with GBS following EBV-associated IM.

## Case presentation

A 19-year-old Caucasian male presented from a referring facility with complaints of generalized weakness involving his upper and lower extremity for one day. Symptoms began with headache, myalgia, and a sensation of numbness and tingling involving the fourth and fifth digits of his upper extremities bilaterally for approximately two weeks. His symptoms worsened and he developed both upper and lower extremity weakness to an extent that he was no longer able to ambulate. His blood pressure was 132/89 mmHg, temperature was 36.4°C (97.6°F), pulse was 78 beats per minute, respiration rate was 20 breaths per minute, and oxygen saturation was 99% on room air. On physical examination, the patient appeared ill, not pale with no icterus. Examination of the oropharynx revealed oropharyngeal exudates, with posterior oropharyngeal erythema and tonsillar hypertrophy. Cervical lymphadenopathy was also noted. On neurological examination, motor strength of the upper and lower extremities was decreased globally with a grade 1 score based on the Medical Research Council muscle strength grading system. Cranial nerves were intact with no speech impairment. Respiratory muscles were also not affected. Deep tendon reflexes were absent globally. Paresthesia was noted globally affecting both upper and lower extremities with a symmetric distribution.

Laboratory findings on presentation were significant for abnormal liver function tests with aspartate aminotransferase of 230 U/L, alanine aminotransferase of 245 U/L, and alkaline phosphatase of 242 U/L. Complete blood count revealed 21.3 K/µL, hemoglobin 14.1 g/dL, and platelets 164 K/µL. White cell differential was notable for 53% lymphocytes, 31% neutrophils, 7% atypical lymphocytes, and 3% bands (Tables 1-3). Lyme titers were within normal limits. HIV antibody test was negative. Serology testing for *Ehrlichia*/*Anaplasma* was negative. Serum antibody titers for cytomegalovirus (CMV), Rickettsia, and hepatitis B and C virus were also negative. The heterophile antibody test was positive. Immunofluorescence assays of antibody titers to EBV antigens showed the presence of Epstein-Barr virus nuclear antigen immunoglobulin (Ig)G antibodies of 131 (0-22), EBVA IgG antibodies of 237 (0-22), and EBVA IgM antibodies of 160 (0-44). A chest X-ray was negative for any acute cardiopulmonary processes.

**Table 1:** Complete blood cell count. WBC: white cell count; RBC: red blood cell count; MCV: mean corpuscular volume; MCH: mean corpuscular hemoglobin; MCHC: mean corpuscular hemoglobin concentration; RDW SD: red cell distribution width standard deviation; RDW CV: red cell distribution width; MPV: mean platelet volume

		Reference range
WBC	21.3 (H)	4.8–10.8 K/µL
RBC	4.81	4.70–6.10 M/µL
Hemoglobulin	14.1	14.0–18.0 g/dL
Hematocrit	42.8	42.0–52.0%
MCV	89.0	80.0–94.0 fL
MCH	29.3	27.0–31.0 pg
MCHC	32.9 (L)	33.0–37.0%
RDW SD	41.1	36.4–46.3 fL
RDW CV	14.4	11.5–14.9 %
Platelets	164	130–400 K/uL
MPV	10.9	9.5–14.4 fL

**Table 2:** Peripheral blood smear findings.

		Reference range
Neutrophils absolute	7.24	2–8.1
Lymphocytes absolute	11.29	0.75–5.5 ×10^3^/µL
Monocytes absolute	0.85	0–1.2 ×10^3^/µL
Eosinophils absolute	0.00	0–0.75 ×10^3^/µL
Basophils absolute	0.43	0–0.4
Neutrophils %	31	42–75%
Lymphocytes %	53	16–52%
Monocytes %	4	0–11%
Eosinophils %	0	0–7%
Basophils %	2	0–4%
Atypical lymphocytes %	7	%
Bands %	3	0–3%

**Table 3:** Chemistry findings. BUN: blood urea nitrogen; AST: aspartate transaminase; ALT: alanine transaminase; ALP: alkaline phosphatase; EGFR: estimated glomerular filtration rate

		Reference range
Sodium	141	135–148 mmol/L
Potassium	4.4	3.5–5.2 mmol/L
CO_2_	22	21–32 mmol/L
Chloride	110	100–110 mmol/L
BUN	9.0	3.0–23.0 mg/dL
Creatinine	0.77	0.80–1.30 mg/dL
Glucose	94	65–99 mg/dL
AST	230	0–48 U/L
ALT	245	13–61 U/L
Total protein	6.7	6.0–8.3 g/dL
Albumin	3.4	3.4–5 g/dL
Total Bilirubin	1.00	0.20–1.00 mg/dL
Calcium	8.7	8.4–10.6 mg/dL
ALP	242	45–136 U/L
A/G ratio	1.03	0.90–2.00
Anion gap	9.0	4.3–12.3 mmol/L
EGFR	131.6	>60.0 mL/minute/1.73m^2^
BUN/creatinine ratio	11.69	10.00–24.00

The patient was eventually admitted to the intensive care unit (ICU) for close monitoring. His negative inspiratory force was -25 with respiratory compromise as a concern. He was subsequently intubated. Lumbar puncture was performed, and cerebrospinal fluid (CSF) revealed a white cell count of 12 cells/mm^3^, red cell count of 7 cells/mm^3^, glucose 53 mg/dL, and elevated protein of 170 mg/dL (Table 4). Serology testing was negative for West Nile virus, syphilis, Lyme disease, and EBV. CSF was significant for albumin-cytologic dissociation consistent with GBS. Motor and sensory nerve conduction studies were not performed. The patient was diagnosed with GBS based on clinical symptoms and CSF findings. He initially received a total of five plasmapheresis treatments, one treatment every other day with no clinical improvement. Given no improvement with plasmapheresis, He received an additional five doses of intravenous immunoglobulin (IVIG). He showed progressive recovery of upper extremity motor and sensory functions. The patient underwent a tracheostomy and was extubated. He was transferred to the medical service on the 14th day of his hospital stay. Motor function of his upper extremities returned to the baseline. He also showed significant improvement in the motor and sensory functions of his lower extremities. The rest of his hospitalization was uneventful, and he was discharged to a rehabilitation facility one month after the presentation.

**Table 4:** CSF findings. CSF: cerebrospinal fluid; WBC: white blood cell; RBC: red blood cell

CSF cell count		
Appearance	Clear	
Color	Colorless	
Tube number	2	
WBC	12	0–5/mm^3^
RBC	7	/mm^3^ undiluted
Segmental cells	23	
Lymphocyte	68	
Monocytes	9	
Total cells counted	100	
CSF chemistry		
Protein	170.0	15.0–45.0 mg/dL
Glucose	53	40–80 mg/dL

## Discussion

This case illustrates the classical features of IM including clinical presentation and heterophile antibody positivity. The typical features of IM include fever, pharyngitis, adenopathy, fatigue, and atypical lymphocytosis [1]. More than half of patients diagnosed with IM present with the triad of fever, pharyngitis, and lymphadenopathy [5]. The laboratory findings commonly seen include lymphocytosis, anemia, elevated aminotransferases, and the presence of heterophile antibodies. Lymphocytosis is the most common finding and can be classified as an absolute count of >4,500/µL or a differential count greater than 50% on peripheral smear.

A peripheral smear helps diagnose IM. Patients with IM may have atypical lymphocytosis, defined as more than 10% of total lymphocytes identified on peripheral smear and is caused by lymphoid cell expansion. This is a hallmark finding of acute IM. These lymphocytes are predominantly T lymphocytes produced to target EBV-infected B lymphocytes [6]. The presence of 10% atypical lymphocytes on peripheral smear raises a high index of suspicion for IM. In a study involving 156 heterophile-positive patients, lymphocytosis of ≥50% was seen in two-thirds, and an atypical lymphocytosis of ≥10% was seen in 75% of the patients. The study reported a sensitivity of 75% and specificity of 92% based on the presence of 10% atypical lymphocytes on peripheral smear [7].

Atypical lymphocytes are non-specific and can be found in other pathologies such as toxoplasmosis, rubella, roseola, viral hepatitis, mumps, CMV, acute HIV infection, and certain drug reactions. For patients who present with typical findings of IM, a peripheral smear helps diagnose IM, especially for patients with cervical lymphadenopathy and unusual presentation. Our patient did not have typical findings described on peripheral smear.

Neurologic complications have been reported with IM, including cranial nerve palsies [2], encephalitis [3], aseptic meningitis, transverse myelitis, peripheral neuritis, optic neuritis, encephalomyelitis, and GBS which has been rarely reported [4]. The most common complications reported are meningitis and meningoencephalitis, with GBS as an uncommon complication. Although the exact etiology of GBS is unknown, a proposed mechanism is an antecedent infection evoking an immune response, which, in turn, results in molecular mimicry [8]. Approximately two-thirds of patients provide a history of an antecedent respiratory tract or gastrointestinal infection [9]. The common pathogens reported include *Campylobacter jejuni*, CMV, EBV, *Mycoplasma*, influenza, and HIV [10,11].

EBV is a herpesvirus and, like other members of the herpesvirus family, has a latency phase. It is the etiologic agent of IM. The pathophysiology of EBV-associated GBS is not clearly understood. However, EBV has been known to have a predilection for B lymphocytes. This results in polyclonal B cell activation, which, in turn, leads to increased Ig production. EBV-induced GBS results in the demyelinating subtype of GBS [8]. Positive heterophile antibodies in a patient with a typical presentation for EBV indicate a diagnosis of EBV infection. Consequently, further testing for specific antibodies to EBV is not necessary for patients with a reactive heterophile antibody. Although EBV-specific antibodies are increasingly used for diagnosis, heterophile results typically return more quickly. Thus, the heterophile test remains the diagnostic point-of-care test of choice in many clinical settings [12]. In our case, the patient had elevated EBV-specific antibodies confirming that he had primary EBV infection.

A case-control study from the Netherlands evaluated 308 patients for serologic evidence of infection with 16 agents and found that recent infection with *C. jejuni*, CMV, and EBV was significantly more common on multivariate analysis among patients who developed GBS than among matched control patients who had other neurologic diseases [13]. The incidence of GBS after an EBV infection was reported to be approximately 10% [14]. Grose et al. described five patients with GBS, aged 18 months to 27 years, with very high levels of antibodies to EBV. Two of the five patients had IM but three had neither clinical nor laboratory evidence of the disease. They concluded that there was enough evidence to suggest an association of EBV with GBS even without signs of IM [14].

GBS is an acute monophasic illness that causes rapidly progressive polyneuropathy with weakness or paralysis. The cardinal clinical features of GBS are progressive, mostly ascending symmetric muscle weakness and absent or depressed deep tendon reflexes [15]. Initial diagnosis is based on clinical presentation, detailed history, and physical examination. The clinical diagnosis of GBS is supported if CSF and electrodiagnostic studies show typical abnormalities [16]. CSF analysis reveals an elevated CSF protein with a normal CSF white blood cell count. This finding, known as albumin-cytologic dissociation, is present in 50-66% of patients with GBS in the first week after the onset of symptoms and ≥75% of patients in the third week [8]. Electrodiagnostic studies such as nerve conduction studies and needle electromyography (EMG) are valuable for confirming the diagnosis of GBS and provide information regarding prognosis. Typical findings include decreased motor nerve conduction velocity and decreased distal motor/sensory amplitudes with a predominately demyelinating pattern [17]. These findings can be normal early during GBS and are typically most pronounced approximately two weeks after the onset of weakness [17]. In our case, the diagnosis was made based on clinical findings and was supported by CSF findings.

Treatment for GBS includes supportive therapy and immunotherapy regardless of its etiology because approximately 30% of patients develop neuromuscular respiratory failure requiring mechanical ventilation [18]. Autonomic dysfunction may be severe with patients requiring ICU monitoring. Our patient was intubated as he was at high risk of respiratory failure. The main treatment modalities for GBS include plasmapheresis and administration of IVIG. These treatments hasten recovery from GBS [19]. Patients recover faster when treated early. Both plasma exchange and IVIG have been reported to have equal efficacy in shortening recovery time and duration of ventilation [19]. The treatment decision between plasma exchange and IVIG depends solely on factors such as availability, patient preference, and contraindications. Our patient received both plasmapheresis and IVIG before showing improvement. Prognosis is good based on reports with full recovery of motor strength in about 60% of patients. Approximately 5-10% of patients have a prolonged course with very delayed and incomplete recovery, and 3-7% die despite receiving intensive care [20].

## Conclusions

This is a rare case of EBV-associated IM GBS. IM is a self-limiting disease but can cause systemic complications, with GBS being one of the known but rare complications. Neurological complications have been reported in approximately 2% of patients. Only a few cases of IM leading to GBS have been reported in the literature. Given its rare occurrence, physicians might be at risk of missing the association between IM and GBS. Detailed history and physical examination can help identify patients with IM-induced GBS. Moreover, increased awareness can help physicians easily identify and manage GBS enabling timely recognition and initiation of prompt supportive care to improve recovery time.
